# First Report of FARSA in the Regulation of Cell Cycle and Survival in Mantle Cell Lymphoma Cells via PI3K-AKT and FOXO1-RAG1 Axes

**DOI:** 10.3390/ijms24021608

**Published:** 2023-01-13

**Authors:** Min Feng, Kun Yang, Jia Wang, Guilan Li, Han Zhang

**Affiliations:** 1 Institute of Medical Biology, Chinese Academy of Medical Sciences and Peking Union Medical College, Kunming 650118, China; 2 School of Life Sciences, Yunnan University, Kunming 650500, China

**Keywords:** mantle cell lymphoma, phenylalanyl-tRNA synthetase subunit alpha, cell cycle, cell apoptosis, regulatory network, forkhead box O1

## Abstract

Cancer-associated factors have been largely identified in the understanding of tumorigenesis and progression. However, aminoacyl-transfer RNA (tRNA) synthetases (aaRSs) have so far been neglected in cancer research due to their canonical activities in protein translation and synthesis. FARSA, the alpha subunit of the phenylalanyl-tRNA synthetase is elevated across many cancer types, but its function in mantle cell lymphoma (MCL) remains undetermined. Herein, we found the lowest levels of *FARSA* in patients with MCL compared with other subtypes of lymphomas, and the same lower levels of FARSA were observed in chemoresistant MCL cell lines. Unexpectedly, despite the essential catalytic roles of FARSA, knockdown of FARSA in MCL cells did not lead to cell death but resulted in accelerated cell proliferation and cell cycle, whereas overexpression of FARSA induced remarkable cell-cycle arrest and overwhelming apoptosis. Further RNA sequencing (RNA-seq) analysis and validation experiments confirmed a strong connection between FARSA and cell cycle in MCL cells. Importantly, FARSA leads to the alteration of cell cycle and survival via both PI3K-AKT and FOXO1-RAG1 axes, highlighting a FARSA-mediated regulatory network in MCL cells. Our findings, for the first time, reveal the noncanonical roles of FARSA in MCL cells, and provide novel insights into understanding the pathogenesis and progression of B-cell malignancies.

## 1. Introduction

Aminoacyl-transfer RNA (tRNA) synthetases (aaRSs) belong to an ancient enzyme family that ligate specific amino acids to their cognate tRNAs for protein synthesis. In mammalian cells, all aaRSs, except for alanyl-tRNA synthetase, append new non-catalytic domains or motifs during evolution, such as the WHEP domains, the glutathione S-transferase domains, etc. These additional domains or motifs allow aaRSs to expand diverse functions beyond its catalytic roles in multiple human diseases, including cancers [1,2]. In humans, the cytoplasmic (cyto) protein synthesis is mediated by cyto-aaRSs, which are encoded by the *AARS1* genes. Interestingly, nearly all cyto-aaRSs are involved in various signaling pathways [1,3,4,5,6], the dysfunction of which is a hallmark of cancer. Therefore, it is not surprising that cyto-aaRSs have been recognized as key participants in tumorigenesis. For example, threonyl-tRNA synthetase 1 (*TARS1*), glycyl-tRNA synthetase 1 (*GARS1*), glutamyl-prolyl-tRNA synthetase 1 (*EPRS1*), and phenylalanyl-tRNA synthetase subunit alpha (*FARSA*) are differentially upregulated across many cancer types, whereas a few cyto-aaRSs, including tryptophanyl-tRNA synthetase 1 (*WARS1*) and isoleucyl-tRNA synthetase 1 (*IARS1*), are downregulated, suggesting that these cyto-aaRSs possess certain specific roles in tumorigenesis [2].

Among these, phenylalanyl-tRNA synthetase (FARS, also termed PheRS) is a typically heterotetrametric protein consisting of two alpha and two beta subunits encoded by *FARSA* and *FARSB*, respectively. Therefore, cyto-PheRS is one of the most complex members of the aaRSs family. Although both alpha and beta subunits are necessary for the enzymatic activity of PheRS, each subunit behaves quite differently with independent roles. For instance, the transcriptional profile of *FARSA* is distinct from that of *FARSB* across different cancer types [2]. In *Drosophila*, FARSA regulates cell growth and proliferation independently of the FARSB subunit and of the aminoacylation activity [7]. The association between FARSA and the tumorigenic events has also been previously reported in human myeloid leukemia cell lines [8]. These findings suggest that FARSA has gained essential non-redundant roles during evolution. Nonetheless, the extra-translational roles of FARSA in B-cell malignances remain unknown.

Mantle-cell lymphoma (MCL) is an aggressive subtype of B-cell lymphoma, accounting for about 6% of non-Hodgkin’s lymphomas (NHLs). Despite improved therapeutic responses with new agents, patients with MCL often relapse with short survival and poor prognosis [9,10]. One major reason is the cellular heterogeneity of MCL, which makes patients highly refractory to standard radiation and chemotherapy. Although the overexpression of cyclin D1 (CCND1), caused by the t(11;14) (q13;q32) translocation is a major hallmark for MCL, some patients lack this translocation, and approximately 2% of healthy individuals harbor it [11], suggesting that other key factors and pathways are required for the pathogenesis and progression of MCL.

Given that the moonlight roles of FARSA have so far remained undetermined in MCL, we started with the analysis of publicly available transcriptomic data on patients with NHLs and MCL, followed by the observation of phenotypes using the FARSA-manipulated MCL cell lines. We further performed RNA sequencing (RNA-seq) analysis and validation experiments to delineate the downstream signaling pathways mediated by FARSA. Based on these data, we revealed an unexpected tumor-suppressing role of FARSA and provided evidence of the FARSA-mediated regulatory network in MCL.

## 2. Results

### 2.1. Expression Feature of FARSA in NHLs and MCL Cell Lines

**Figure 1:**
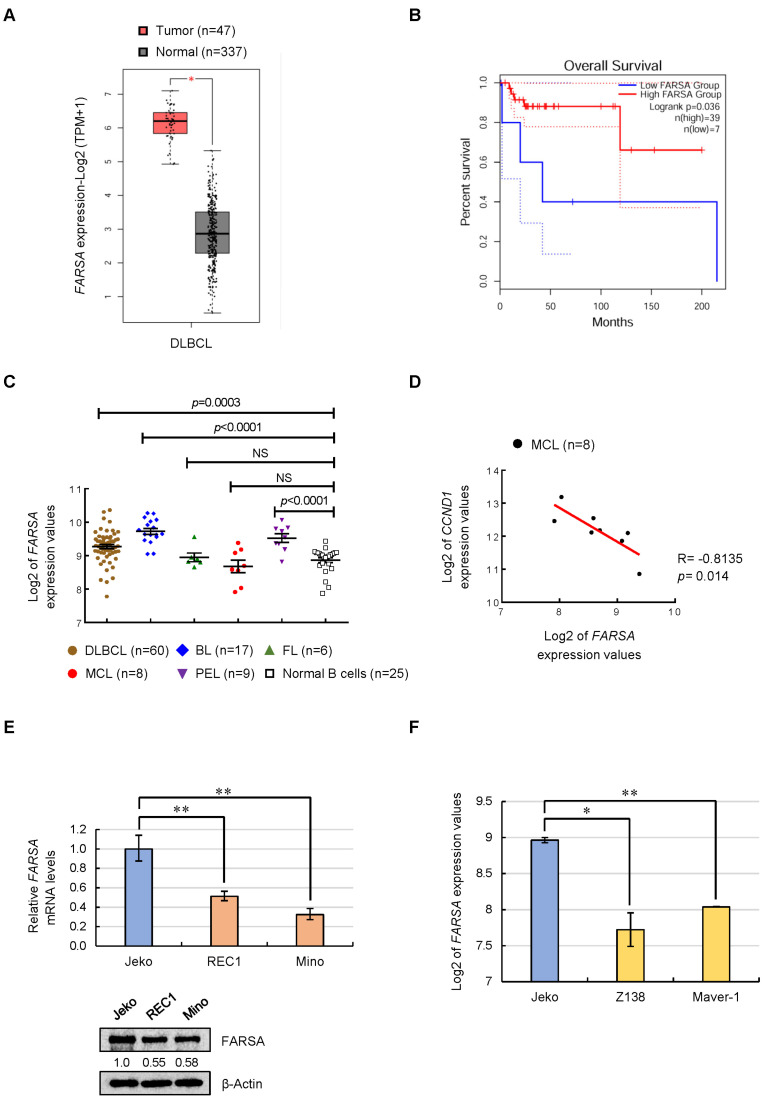
Expression feature of phenylalanyl-tRNA synthetase subunit alpha (FARSA) in non-Hodgkin’s lymphomas (NHLs) and mantle cell lymphoma (MCL) cell lines. (**A**) *FARSA* mRNA levels in diffuse large B-cell lymphoma (DLBCL, red box plot) and normal tissues (grey box plot) using GEPIA2 generated by the Cancer Genome Atlas (TCGA) data. (**B**) The survival curve of patients with DLBCL was generated with the TCGA database using the cutoff-high (15%) and cutoff-low (15%) levels of mRNA to separate patients into a high expression group and a low expression group, respectively, for *FARSA*. (**C**) *FARSA* mRNA levels in patients with MCL (n = 8) compared with patients with other subtypes including DLBCL (n = 60), Burkitt lymphoma (BL, n = 17), follicular lymphoma (FL, n = 6) and primary effusion lymphoma (PEL, n = 9) as well as the normal B cells (n = 25). Log2 of the *FARSA* expression values are shown as the mean ± standard error of mean (SEM) based on a micro-array dataset (GSE2350). (**D**) Correlation analysis of *FARSA* and cyclin D1 (*CCND1*) mRNA expression using the log2 of their expression values based on a micro-array dataset (GSE2350) in patients with MCL (n = 8). R value and *p* value are indicated. (**E**) Relative mRNA levels (**upper**) and immunoblots (**lower**) of FARSA in Jeko, REC1 and Mino MCL cells. Each value from quantitative real-time PCR (qRT-PCR) was run in triplicate with the values normalized to actin beta (*ACTB*). Data are shown as the mean ± standard deviation (SD) relative to the Jeko group. β-Actin was used as a loading control for immunoblots with the normalized values (FARSA/β-Actin) indicated under each lane. (**F**) Log2 of the *FARSA* expression values in Jeko, Z138 and Maver-1 MCL cells based on another micro-array dataset (GSE42549). NS, not significant; * *p* < 0.05; ** *p* < 0.01.

To investigate the expression feature of FARSA in NHLs, we started with analysis of the clinical data on lymphoma tissues using GEPIA2 generated by the Cancer Genome Atlas (TCGA) project. Compared with normal tissues, *FARSA* was upregulated in diffuse large-B cell lymphoma (DLBCL) (Figure 1A), in line with what has been reported [2]. In spite of the upregulation of *FARSA*, DLBCL patients with high *FARSA* at a cutoff-low of 15% showed higher survival rates than patients with low *FARSA* (Figure 1B), implying a tumor suppressor-like role of FARSA in DLBCL.

Because other subtypes of NHLs are not included in the TCGA database, we then used a published micro-array dataset (GSE2350) to analyze the expression feature of *FARSA* among different subtypes. Intriguingly, we found higher *FARSA* levels in patients with DLBCL, Burkitt lymphoma (BL) and primary effusion lymphoma (PEL) compared with normal B cells, whereas no significant differences were observed in *FARSA* levels between MCL and normal B cells, and the same is true regarding follicular lymphoma (FL) (Figure 1C). Nevertheless, it is worth noting that patients with MCL showed the lowest levels of *FARSA* compared with other subtypes. More interestingly, further correlation analysis using the same micro-array dataset revealed a significant negative correlation between *FARSA* and *CCND1* expressions in MCL patients (Figure 1D).

Next, we focused on the MCL and compared the expression levels of FARSA in three MCL cell lines, of which, Jeko cells are sensitive to Bortezomib (BTZ), the first proteasome inhibitor approved for the treatment of relapsed/refractory patients with MCL, whereas REC1 and Mino cells are BTZ-resistant MCL cell lines [12,13]. Of note, we found the highest levels of FARSA in Jeko cells compared with REC1 and Mino cells, both in mRNA and protein levels (Figure 1E). Consistently, based on another micro-array dataset (GSE42549), the MCL cell lines Z138 and Maver-1, which are resistant to pan-protein kinase C inhibitors Sotrastaurin (STN), also displayed lower *FARSA* levels compared with the STN-sensitive Jeko cells (Figure 1F). Altogether, this discovery, along with the low *FARSA* levels in MCL patients (Figure 1C) as well as the negative correlation between *FARSA* and *CCND1* (Figure 1D), suggests that FARSA may serve as a tumor suppressor in MCL, and low FARSA levels may confer chemoresistant properties to MCL cells.

### 2.2. Knockdown of FARSA Accelerates Cell Proliferation and Cell Cycle in FARSA-Higher MCL Cells

**Figure 2:**
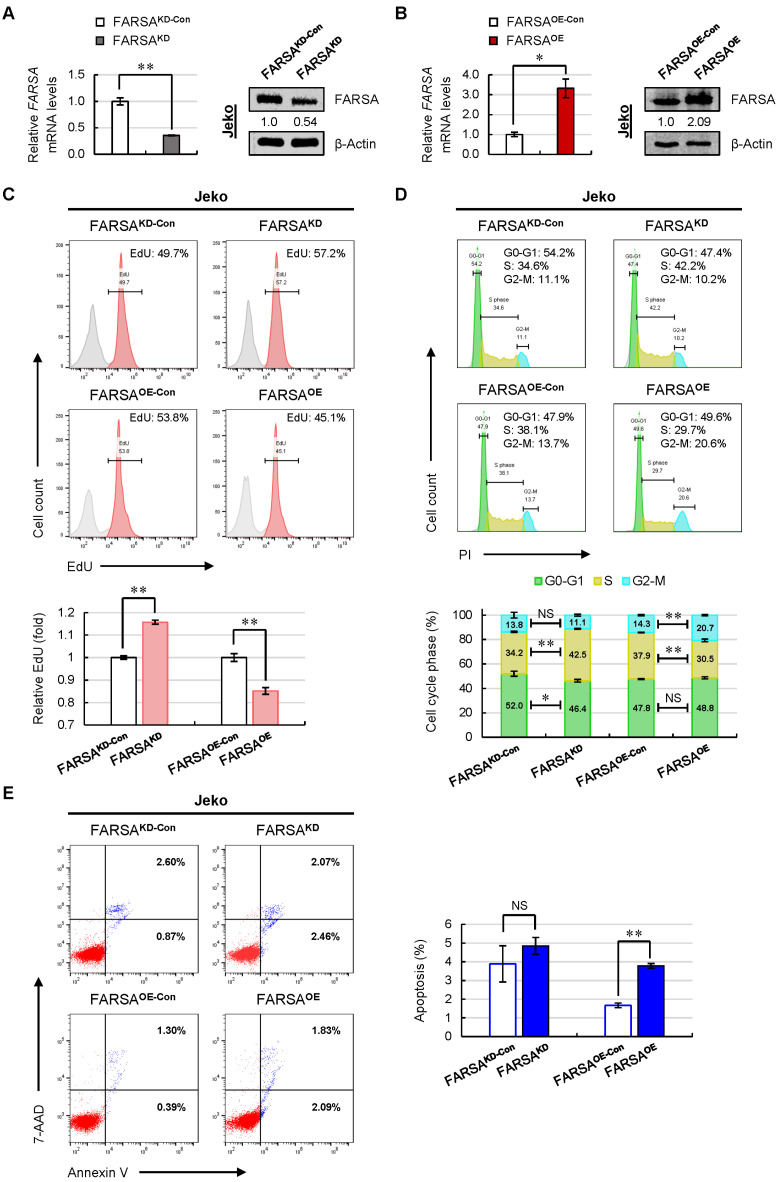
Knockdown of FARSA in Jeko cells accelerates cell proliferation and cell cycle. The knockdown (**A**) and overexpressed efficiency (**B**) of FARSA in Jeko cells were validated using qRT-PCR (**left**) and immunoblotting (**right**), respectively. Each value from qRT-PCR was normalized to *ACTB* and is presented as the mean ± SD. β-Actin was used as a loading control for immunoblots with the normalized values (FARSA/β-Actin) indicated under each lane. (**C**) Representative intracellular pulse staining of EdU in FARSA^KD^ and FARSA^OE^ Jeko cells (**upper**). The % population of EdU positive cells was normalized to the control cells (**lower**), and the data are shown as the mean ± SD from three independent experiments. (**D**) Representative cell cycle distribution of FARSA^KD^ and FARSA^OE^ Jeko cells staining with PI (**upper**). The % population of cells in each phase is shown as the mean ± SD from three independent experiments (**lower**). (**E**) Representative cell apoptosis in FARSA^KD^ and FARSA^OE^ Jeko cells staining with Annexin V/7-AAD (**left**). The % population of apoptotic cells in each group is shown as the mean ± SD from three independent experiments (**right**). NS, not significant; * *p* < 0.05; ** *p* < 0.01 (vs. control group).

To determine the roles of FARSA in MCL, we manipulated FARSA levels in MCL cell lines via lentiviral infection. Given that the complete knockout of FARSA would lead to cell death due to defective and deficient synthesis of protein, we utilized Jeko cells to reduce FARSA (FARSA^KD^) via a lentiviral shRNA-mediated knockdown system, since Jeko cells showed high basal levels of FARSA (Figure 1E). The knockdown efficiency of FARSA was evaluated by quantitative real-time PCR (qRT-PCR) and immunoblots, which showed a decrease of FARSA expression by approximately half (Figure 2A). Meanwhile, we generated stable FARSA-overexpressing (FARSA^OE^) Jeko cells in parallel (Figure 2B).

Compared with control cells, intracellular pulse staining for EdU incorporation showed higher amounts of proliferated cells upon FARSA silencing and lower amounts of proliferated cells after FARSA overexpression in Jeko cells (Figure 2C). Further analysis of cell-cycle distribution confirmed approximately 8% more cells in S phase in FARSA^KD^ cells compared with control cells, whereas the diminished proliferation in FARSA^OE^ cells was associated with decreased S-phase population (Figure 2D). We next questioned the effects of FARSA levels on cell survival. As shown in Figure 2E, a comparable proportion of apoptotic cells was observed for FARSA^KD^ and control cells, indicating that knockdown of FARSA did not affect cell survival in MCL; however, overexpression of FARSA led to a significant increase of cell apoptosis. These data indicate that reducing FARSA levels in Jeko cells did not inhibit cell growth but led to increased cell proliferation and accelerated cell cycle, whereas overexpression of FARSA showed the opposite effects in cell proliferation and cell cycle and triggered apoptosis.

### 2.3. Overexpression of FARSA Induces Remarkable Cell-Cycle Arrest and Apoptosis in FARSA-Lower MCL Cells

**Figure 3:**
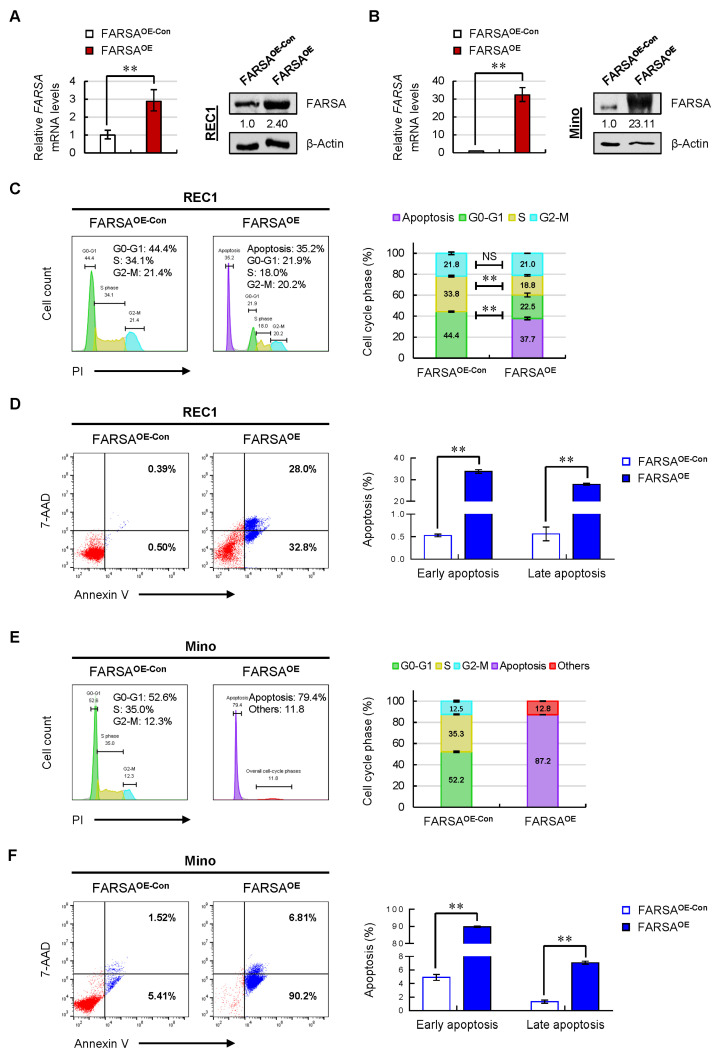
Overexpression of FARSA in REC1 and Mino cells induces remarkable cell-cycle arrest and overwhelming cell apoptosis. The overexpressed efficiency of FARSA in REC1 (**A**) and Mino (**B**) cells were validated using qRT-PCR (**left**) and immunoblotting (**right**), respectively. Each value from qRT-PCR was normalized to *ACTB* and is presented as the mean ± SD. β-Actin was used as a loading control for immunoblots with the normalized values (FARSA/β-Actin) indicated under each lane. (**C**) Representative cell-cycle distribution of FARSA^OE^ REC1 cells staining with PI (**left**). The % population of cells in each phase is shown as the mean ± SD from three independent experiments (**right**). (**D**) Representative cell apoptosis in FARSA^OE^ REC1 cells staining with Annexin V/7-AAD (**left**). The % population of early- and late-apoptotic cells in each group is shown as the mean ± SD from three independent experiments (**right**). (**E**) Representative cell-cycle distribution of FARSA^OE^ Mino cells staining with PI (**left**). The % population of cells in each phase is shown as the mean ± SD from three independent experiments (**right**). (**F**) Representative cell apoptosis in FARSA^OE^ Mino cells staining with Annexin V/7-AAD (**left**). The % population of early- and late-apoptotic cells in each group is shown as the mean ± SD from three independent experiments (**right**). NS, not significant; ** *p* < 0.01 (vs. control group).

To validate our observations in Jeko cells, we further overexpressed FARSA in REC1 (Figure 3A) and Mino (Figure 3B) MCL cells. Considering that the REC1 and Mino cells have low basal levels of FARSA (Figure 1E), they should be more sensitive to FARSA overexpression than Jeko cells. Indeed, overexpression of FARSA in REC1 cells led to an overall cell-cycle arrest in G0–G1 and S phases accompanied by a significant elevation of apoptotic peak (Figure 3C). In addition, FARSA^OE^ REC1 cells also underwent an overwhelming apoptosis compared with control cells, both in early and late phases (Figure 3D), suggesting that the low levels of FARSA in REC1 cells are critical in maintaining MCL survival.

Notably, we observed a dose-dependent effect due to the dependence of the cellular phenotypes on the degree of FARSA overexpression. Compared with FARSA^OE^ REC1 cells, more overwhelming cell-cycle arrests and apoptoses were triggered in FARSA^OE^ Mino cells (Figure 3E, F), which showed an over 20-fold increase of FARSA (Figure 3B). This effect ultimately resulted in a rapid cell death of the FARSA^OE^ Mino cells. Collectively, the phenotypes observed in the manipulated MCL cells further supported a tumor suppressor-like role of FARSA in MCL cells. More importantly, these data suggest that there is a fine control of the FARSA-mediated regulatory network in MCL. Once the balance of FARSA levels is broken, especially in the lower FARSA MCL cells, there will emerge a disordered cell cycle and cell survival.

### 2.4. RNA-Seq Profiles of FARSA^KD^ Jeko Cells Reveal Noncanonical Roles of FARSA in MCL

**Figure 4:**
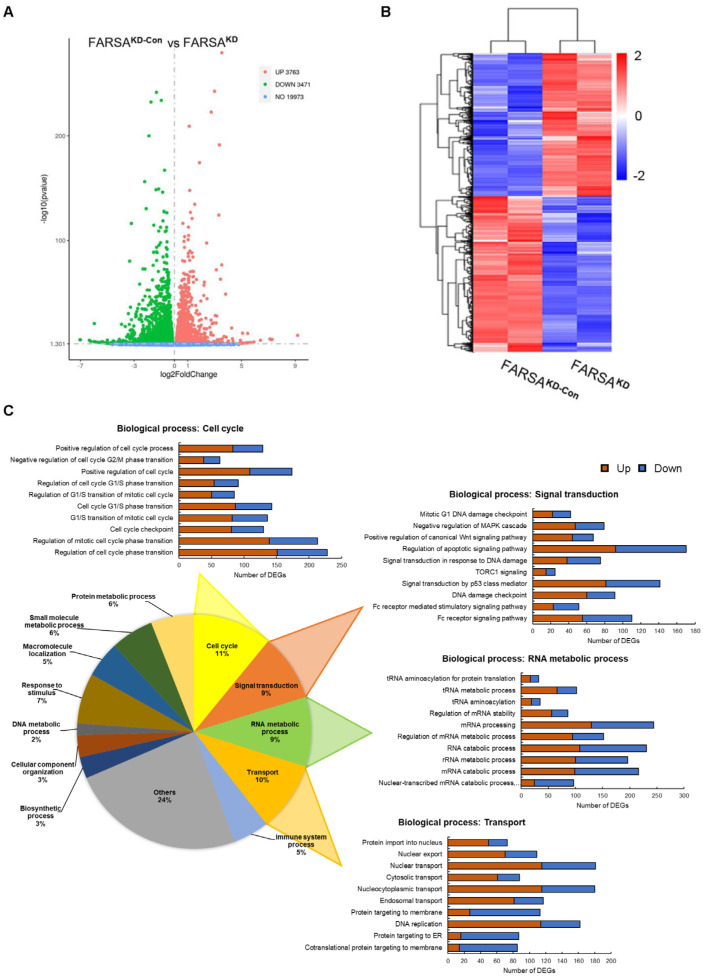
The differentially expressed genes (DEGs) and the Gene Ontology (GO) terms enriched in the biological process category affected by FARSA in Jeko cells. (**A**) Volcano plots of RNA sequencing (RNA-seq) data in FARSA^KD^ Jeko cells relative to the control cells. Green dots represent the downregulated DEGs (n = 3471) and red dots represent the upregulated DEGs (n = 3763), whereas blue dots (n = 19973) represent genes not significantly altered. (**B**) Heatmap of RNA-seq data from independent samples (n = 2/group) of FARSA^KD^ and FARSA^KD-Con^ Jeko cells. The fold change in gene expression is indicated by the color intensity, with blue representing the downregulated DEGs and red representing the upregulated DEGs. (**C**) The pie chart displaying the sub-categories of the GO terms enriched in the “biological process” category based on the AmiGO 2 software. The detailed GO terms in four representative sub-categories are shown around the pie chart, with red representing the upregulated DEGs and blue representing the downregulated DEGs.

Because FARSA^KD^ Jeko cells showed an oncogenic feature with good viability, we further utilized RNA-seq to capture the transcriptome changes mediated by FARSA in Jeko cells. Total mRNA from the FARSA^KD^ Jeko cells was compared with the control group, which displayed 7234 differentially expressed genes (DEGs) including 3763 upregulated DEGs and 3471 downregulated DEGs (Figure 4A). The resulting heatmap shows two hierarchical clusters based on similar gene expression profiles from two replicates of two different settings (Figure 4B).

We next performed Gene Ontology (GO) analysis to identify potential molecular mechanisms of FARSA in MCL cells. On the basis of the obtained significant GO terms, we utilized the AmiGO 2 tool to classify them into sub-categories. Intriguingly, in the biological process category, approximately 11% of enriched terms were classified into “Cell cycle” (Figure 4C), in line with the cellular phenotypes. In addition, nearly 20% of enriched terms were classified into “Signal transduction” and “Transport”, most of which are correlated with tumorigenesis and progression, such as DNA damage, regulation of cancer-related pathways, and nuclear or cytosolic transport. Although 9% of the enriched GO terms were classified into “RNA metabolic process”, a canonical role of FARSA, the other three sub-categories mainly reflected the noncanonical function of FARSA in MCL. Likewise, in the cellular component category, most enriched GO terms were classified into “Protein-containing complex”, “Cytoplasm” and “Membrane” (Supplementary Figure S1A), whereas in the molecular function category, the enriched terms included “Ras GTPase binding”, “DNA polymerase binding”, “Cell adhesion molecule binding”, etc. (Supplementary Figure S1B), all suggesting the moonlight roles of FARSA in MCL.

**Figure 5:**
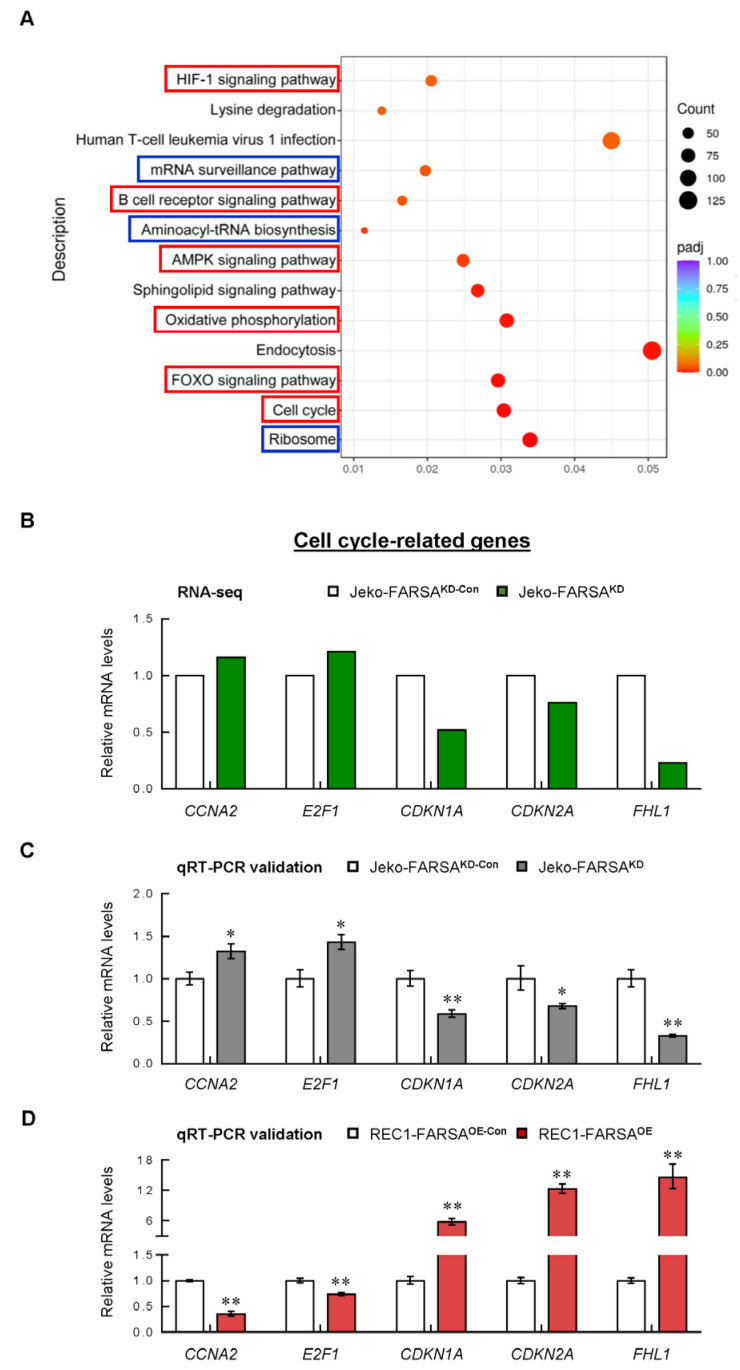
The enriched Kyoto Encyclopedia of Genes and Genomes (KEGG) pathways and validation experiments of cell cycle-related genes. (**A**) KEGG pathway enrichment scatter plot, with blue boxes indicating the canonical pathways and red indicating the cancer-related pathways. The vertical axis represents the pathway name, and the horizontal axis represents the pathway factor corresponding to the Rich factor. The size of the adjusted *p* value is represented by the color of the point. The smaller the adjusted *p* value, the closer the color is toward red. The number of the DEGs included in each pathway are expressed by the size of the point. (**B**) Relative mRNA levels of the selected cell cycle-related DEGs from the RNA-seq. Each value from the RNA-seq in FARSA^KD^ Jeko cells was relative to the control cells. Validation experiments in FARSA^KD^ Jeko cells (**C**) and FARSA^OE^ REC1 cells (**D**). Each value from qRT-PCR was normalized to *ACTB* and is presented as the mean ± SD. * *p* < 0.05; ** *p* < 0.01 (vs. control group).

In the Kyoto Encyclopedia of Genes and Genomes (KEGG) analyses, apart from canonical pathways such as “Ribosome”, “Aminoacyl-tRNA biosynthesis” and “mRNA surveillance pathway”, the DEGs were mostly enriched in cancer-related signaling pathways, including “Cell cycle”, “FOXO signaling pathway”, “Oxidative phosphorylation”, “AMPK signaling pathway”, “B cell receptor signaling pathway” and “HIF-1 signaling pathway” (Figure 5A). Among these, “Cell cycle” was once again revealed to be the most enriched signaling, and “FOXO signaling pathway” ranked the second, highlighting their importance in FARSA-manipulated MCL cells.

### 2.5. Cell Cycle- and Cancer-Related Genes Are Significantly Altered in FARSA-Manipulated MCL Cells

We next selected the most differentially changed cell cycle-related genes that are closely associated with MCL. These DEGs included cell-cycle activators cyclin A2 (*CCNA2*) and E2F transcription factor 1 (*E2F1*), as well as cell-cycle inhibitors cyclin dependent kinase inhibitor (CDKN) 1A (*CDKN1A*), *CDKN2A* and four and a half LIM domains 1 (*FHL1*). Among these, CCNA2 is synthesized at the onset of the S phase and during G2/M transition [14]. Elevated CCNA2 expression is associated with high proliferation and poor survival in MCL [15,16]. In addition, D-cyclins bind and activate cyclin dependent kinase (CDK) 4 and/or CDK6 to inactivate RB transcriptional corepressor 1 during cell-cycle progression, allowing E2F1-mediated cell cycle progression and DNA synthesis [17]. Thus, abrogation of E2F1-mediated transcription induces MCL cell death [18]. By contrast, *CDKN1A* and *CDKN2A* encode p21^Cip1^ and p16^Ink4a^, respectively, which are potent cell-cycle inhibitors in MCL [19,20], whereas FHL1 has been revealed to induce G1 and G2/M cell-cycle arrest by activating CDKN1A in human cancers [21,22].

Consistent with the RNA-seq data (Figure 5B), we confirmed the upregulation of *CCNA2* and *E2F1* as well as the downregulation of *CDKN1A*, *CDKN2A* and *FHL1* in FARSA^KD^ Jeko cells compared with control cells (Figure 5C). Further analysis using the FARSA^OE^ REC1 cells exhibited the opposite expression modes (Figure 5D), indicating that overexpression of FARSA reverses the levels of these genes. As such, it is very likely that the strong phenotypes of the cell cycle observed in the manipulated MCL cells are mainly mediated by these genes.

It is of interest to note that multiple cancer-related genes were also significantly increased in FARSA^KD^ Jeko cells, including activating transcription factor 5 (*ATF5*), eukaryotic translation initiation factor 4 gamma 1 (*EIF4G1*), telomerase reverse transcriptase (*TERT*), KRAS proto-oncogene (*KRAS*), mitogen-activated protein kinase (MAPK) kinase 1 (*MAP2K1*) and *MAPK1*, which are key regulators contributing to the development and progression of MCL and other NHLs [12,13,23,24,25] (Supplementary Figure S2). This finding suggests that other mechanisms may be simultaneously involved in FARSA-mediated pathogenesis and progression of MCL in addition to cell cycle.

### 2.6. FARSA-Mediated Enhancement of PI3K-AKT Signaling in MCL Cells

**Figure 6:**
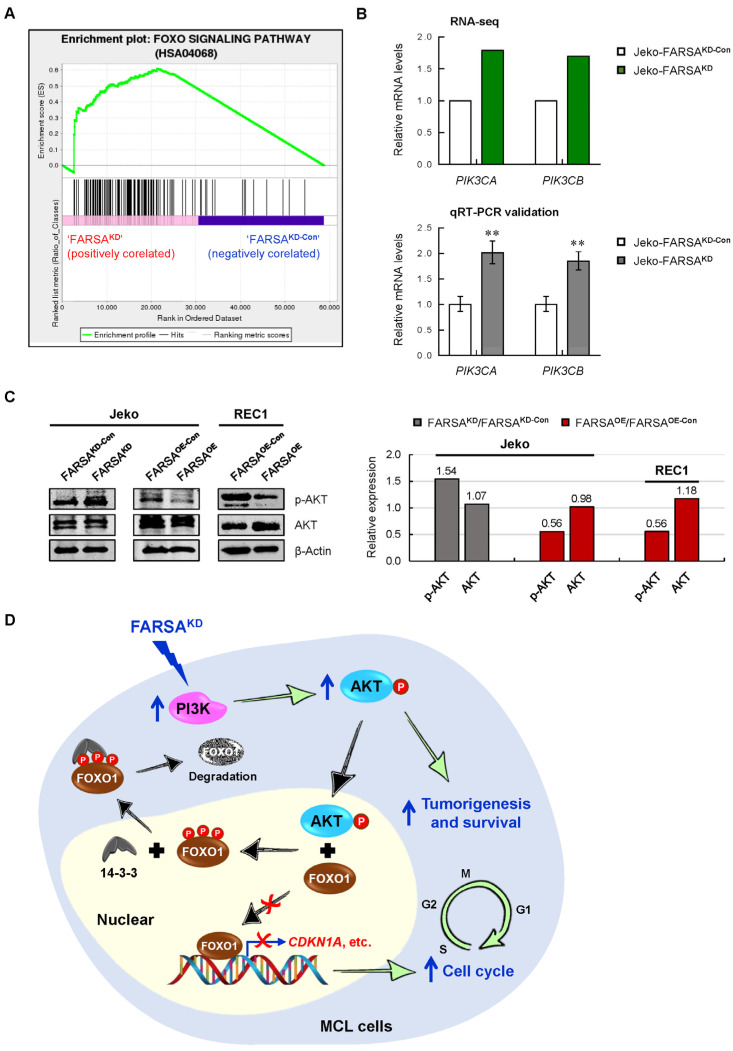
FARSA-mediated enhancement of PI3K-AKT signaling in MCL cells. (**A**) Enriched forkhead box O (FOXO) signaling pathway determined by Gene Set Enrichment Analysis (GSEA). (**B**) Relative mRNA levels of phosphatidylinositol-4,5-bisphosphaste 3-kinase catalytic (PIK3C) subunit alpha (*PIK3CA*) and PIK3C subunit beta (*PIK3CB*) from the RNA-seq (**upper**) and qRT-PCR (**lower**). Each value from the RNA-seq in FARSA^KD^ Jeko cells was relative to the control cells. Each value from qRT-PCR was normalized to *ACTB* and is presented as the mean ± SD. ** *p* < 0.01 (vs. control group). (**C**) Immunoblots of phosphorylated AKT (p-AKT) and AKT in FARSA^KD^ Jeko, FARSA^OE^ Jeko and FARSA^OE^ REC1 cells (**left**). β-Actin was used as a loading control. The expression of each protein was normalized to the corresponding β-Actin, and the relative expression of each protein in FARSA^KD^ or FARSA^OE^ cells is indicated relative to the control cells (**right**). (**D**) FARSA knockdown enhances the activation of PI3K-AKT signaling. AKT-mediated FOXO1 phosphorylation enables its binding to 14-3-3 proteins and nuclear export, leading to FOXO1 degradation. Both the enhanced activation of AKT and cell cycle acceleration caused by AKT-mediated FOXO1 inactivation ultimately contribute to the oncogenic phenotypes of the FARSA^KD^ MCL cells.

The alteration of the cell cycle represents a late effect in cancer cells. We then questioned which pathway is responsible for the regulation of the cell cycle in the FARSA-manipulated MCL cells. Although multiple cancer-related pathways were identified, the forkhead box O (FOXO) signaling pathway ranked higher than the others (Figure 5A). In addition, Gene Set Enrichment Analysis (GSEA) confirmed the upregulation of the FOXO pathway in FARSA^KD^ MCL cells (Figure 6A), highlighting the importance of FOXO signaling in the FARSA-mediated network. Notably, among four human FOXO members, FOXO1 was shown to be critical for B cell development [26,27], and to regulate the genes involved in cell-cycle arrest. The activity of FOXO1 is regulated by PI3K-AKT signaling. Phosphorylation of FOXO1 by AKT leads to its subsequent protein degradation, resulting in an accelerated cell cycle [28]. Given that the PI3K-AKT signaling is constitutively activated in MCL [29,30], we thus reasoned that PI3K-AKT signaling is responsible for the alteration of the cell cycle by inactivating FOXO1 in the FARSA-manipulated MCL cells.

As expected, we found that phosphatidylinositol-4,5-bisphosphaste 3-kinase catalytic (PIK3C) subunit alpha (*PIK3CA*) and PIK3C subunit beta (*PIK3CB*), which encode the alpha and beta catalytic subunits of PI3K, were significantly upregulated in FARSA^KD^ cells, indicating an increase of the catalytic subunits of PI3K (Figure 6B). Further immunoblots confirmed the increased levels of phosphorylated AKT (p-AKT) in FARSA^KD^ Jeko cells, with no significant changes in the expression of total AKT proteins between the two groups; in contrast, decreased p-AKT proteins were observed in Jeko and REC1 cells upon FARSA overexpression (Figure 6C). These findings support a hypothetic molecular event in the FARSA^KD^ MCL cells (Figure 6D), in which knockdown of FARSA enhances the PI3K-AKT signaling, contributing to the accelerated cell cycle by inactivating FOXO1; on the other hand, the enhanced activation of AKT is another contributor to the pathogenesis and survival of MCL [29].

### 2.7. FARSA-Mediated Activation of the FOXO1-RAG1 Signaling in MCL Cells

**Figure 7:**
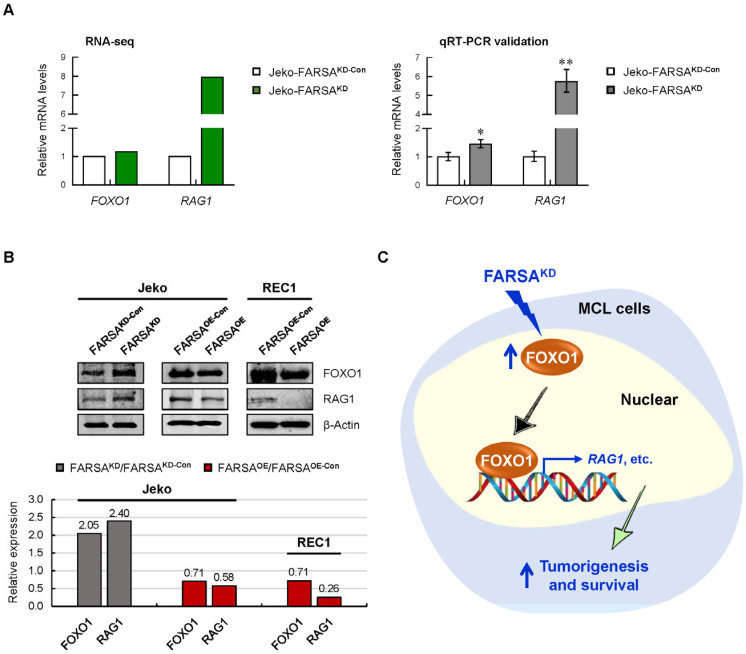
FARSA-mediated activation of the FOXO1-RAG1 signaling in MCL cells. (**A**) Relative mRNA levels of *FOXO1* and recombination activating 1 (*RAG1*) from the RNA-seq (**left**) and qRT-PCR (**right**). Each value from the RNA-seq in FARSA^KD^ Jeko cells was relative to the control cells. Each value from qRT-PCR was normalized to *ACTB* and is presented as the mean ± SD. * *p* < 0.05; ** *p* < 0.01 (vs. control group). (**B**) Immunoblots of FOXO1 and RAG1 in FARSA^KD^ Jeko, FARSA^OE^ Jeko and FARSA^OE^ REC1 cells (**upper**). β-Actin was used as a loading control. The expression of each protein was normalized to the corresponding β-Actin, and the relative expression of each protein in FARSA^KD^ or FARSA^OE^ cells is indicated relative to the control cells (**lower**). (**C**) FARSA knockdown activates the FOXO1-RAG1 signaling which promotes tumorigenesis and survival of MCL cells.

Although the activated PI3K-AKT signaling leads to the degradation of FOXO1 proteins, we surprisingly found a synergistical upregulation of *FOXO1* and recombination activating 1 (*RAG1*) among the DEGs enriched in the FOXO signaling (Figure 7A). This finding implies the activation of FOXO1-RAG1 signaling, another important FOXO1-mediated pathway. Apart from the regulation of cell-cycle arrest, FOXO1 is also a potent transcriptional activator of *RAG1*, which is critical for tumorigenesis and survival in hematological malignancies [31,32,33,34]. Therefore, we hypothesized that the FOXO1-RAG1 signaling is simultaneously activated in MCL cells upon FARSA knockdown. Indeed, the immunoblots confirmed the upregulation of FOXO1 and RAG1 in FARSA^KD^ Jeko cells, and overexpression of FARSA in Jeko and REC1 cells reversed their expression levels (Figure 7B), thus supporting another activated tumor-promoting FOXO1-RAG1 signaling in the FARSA-manipulated MCL cells (Figure 7C).

## 3. Discussion

Although *FARSA* is differentially upregulated across many cancer types (n = 18) [2], the causative connection between the increased FARSA levels and tumorigenesis has so far been overlooked. One major reason might be the assumption that the increased PheRS levels may reflect higher demands of cancer cells for protein synthesis to proliferate and survive. As such, the research on FARSA in human cancer is very limited. While several studies have revealed the moonlighting activity of FARSA outside of aminoacylation [7,8,35], the function of FARSA in MCL cells remains uninvestigated. Here we observed the lowest levels of *FARSA* in patients with MCL compared with other subtypes of NHLs, and the same lower levels of FARSA were found in chemoresistant MCL cell lines. Further knockdown of FARSA in MCL cells did not inhibit but promoted cell proliferation and cell cycle, whereas overexpression of FARSA resulted in the opposite phenotypes. The following GO and KEGG analyses as well as the validation experiments further demonstrated a strong connection between FARSA and cell cycle. All these findings pinpoint the way in which FARSA exerts an anti-tumor effect by modulating the cell cycle. Of note, the survival curve generated with TCGA data indicates the favorable role of *FARSA* in DLBCL patients, and the same is true in patients with kidney renal clear-cell carcinoma [2]. However, the impact of FARSA on MCL patient survival remains unknown. Thus, the function of FARSA expression level as a prognostic factor or, in particular, as a biomarker of sensitivity to anti-MCL agents will need to be further evaluated.

Strikingly, we observed an overwhelming cell apoptosis in REC1 and Mino cells upon FARSA overexpression. One possible interpretation is that the chemoresistant MCL cells with low basal levels of FARSA remain in a metabolically indolent state, which enables their survival under conditions of starvation or hypoxia. In this context, overexpression of FARSA may drive excessive protein synthesis, leading to imbalanced protein homeostasis and aberrant signaling, which are highly toxic to cells. This interpretation is supported by a recent study demonstrating that increased FARSA levels impair insulin signaling by modifying the beta subunit of insulin receptor, reflecting a toxic role of FARSA overexpression [35]. On the other hand, our findings, that knockdown of FARSA leads to an accelerated cell cycle, might be due to the adaption to defective protein synthesis by stimulating other pathways.

Indeed, the DEGs from RNA-seq were enriched in multiple cancer-related signaling pathways, of which the FOXO signaling pathway arouses our great interest. In humans, there are four FOXO family members including FOXO1, FOXO3, FOXO4 and FOXO6, of which FOXO1 is critical for B-cell development [26,27]. FOXO1 regulates the genes involved in cell-cycle arrest, cell death and cell metabolism, and its activity is mainly regulated by PI3K-AKT signaling [28]. AKT-mediated phosphorylation of FOXO1 enables its binding to 14-3-3 proteins and nuclear export, ultimately leading to inactivation. Therefore, FOXO1 acts as a tumor suppressor in various solid tumors and classical Hodgkin lymphoma [36,37,38]. On the contrary, despite phosphorylation, the retained nuclear FOXO1 is a potent transcriptional activator of *RAG1*, *RAG2* and *AICDA*, which are critical for tumorigenesis in B-cell leukemias [31,32]. Another mechanism of FOXO1-driven leukemogenesis has been revealed by a study indicating that the genetic and pharmacological inactivation of FOXO1 induces a strong anti-leukemic effect associated with the downregulation of CCND3, MYC proto-oncogene, bHLH transcription factor (MYC) and mammalian target of rapamycin complex 1 (mTORC1) [33]. In BL, although the PI3K-AKT pathway is activated, there are abundant nuclear FOXO1 proteins which promote tumor growth and survival, presenting as an important oncogenic event in B-cell lymphomagenesis [34]. These findings contradict the tumor suppressor role of FOXO1, reflecting a context-specific feature of FOXO1 in cancer.

In the present study, we found the increased mRNA levels of *FOXO1* and *RAG1* in FARSA^KD^ Jeko cells. Immunoblots further proved the involvement of FARSA-mediated FOXO1-RAG1 axis in MCL cells. The implication of this finding supports a hypothetic model in which knockdown of FARSA leads to increased FOXO1, which elicits an oncogenic effect on MCL cells by activating the downstream RAG1. On the other hand, given the mutual exclusion of PI3K-AKT activation and nuclear FOXO1, the enhanced proliferation and cell cycle in FARSA^KD^ cells might be attributed to the AKT-mediated FOXO1 inactivation. Nevertheless, we cannot exclude the possibility that other pathways or factors are concurrently involved in the FARSA-mediated regulatory network in MCL, since multiple cancer-related pathways were identified based on KEGG analysis, such as oxidative phosphorylation, AMPK signaling, etc. This result highlights the complexity of the FARSA-mediated regulation in MCL, a topic certainly requiring further work.

In fact, FARSA is not the only aaRS with noncanonical functions in human cancer. Increasing evidence has shown the links between aaRSs and tumorigenesis, though this is not well-established [1,2]. For example, WARS1 is associated with the progression and prognosis of solid tumors [39,40,41], designating it as a promising cancer marker and a therapeutic candidate. EPRS1 [3], methionyl-tRNA synthetase 1 [4] and lysyl-tRNA synthetase 1 [5] also play essential roles in the progression and metastasis of cancer cells by regulating different signals. These unexpected aaRS-mediated regulatory networks not only provide novel insights into the tumorigenesis and progression, but also bring a potential clinical strategy by targeting aaRSs or their downstream factors, thus facilitating treatment discovery and development.

Taken together, our study for the first time revealed a tumor-suppressor role of FARSA in MCL cells. Importantly, FARSA leads to the alteration of cell cycle and survival via a tight regulation of PI3K-AKT and FOXO1-RAG1 signaling in MCL cells. Despite these findings, several issues remain unaddressed. For example, do the findings in MCL apply to other B-cell lymphomas or leukemias? How does FARSA alter FOXO1 levels? What are the mechanisms underlying the FARSA-mediated regulation of PI3K-AKT and FOXO1-RAG1 signaling? These answers will lead us to a comprehensive understanding of the noncanonical roles of FARSA in B-cell malignancies.

## 4. Materials and Methods

### 4.1. Clinical Data Sources

Public GEPIA2 and GEO databases were utilized for clinical data analysis in lymphomas. Briefly, GEPIA2 databases on cancer tissues are generated by the TCGA project (https://www.cancer.gov/tcga, accessed on 17 March 2022) and normal tissues are generated by the Genotype-Tissue Expression project. The micro-array data from normal B cells (n = 25) and 100 patients with lymphomas including DLBCL (n = 60), BL (n = 17), FL (n = 6), MCL (n = 8) and PEL (n = 9) [42], as well as the micro-array data from 4 MCL cell lines [43] were downloaded from the GEO databases of GSE2350 and GSE42549, respectively. The log2 of mRNA expression values for *FARSA* and *CCND1* were used for analysis.

### 4.2. Cell Lines and Culture

Three MCL cell lines were used in this study: human MCL cell line Jeko was purchased from the Center of Cell Resources in the Shanghai Institute of Biochemistry and Cell Biology (Shanghai, China), and was authenticated using short tandem repeats (STR) at Cobioer Biosciences (Nanjing, China); the human MCL cell line REC1 was purchased from BNCC (Beijing, China), and was authenticated using STR at CinoAsia Institute (Shanghai, China); human MCL cell line Mino was purchased and authenticated at Cobioer Biosciences (Nanjing, China). Cells were maintained under 5% CO_2_ at 37 °C and cultured in RPMI1640 medium supplemented with 10% fetal bovine serum, and 100 U.I./mL penicillin-streptomycin.

### 4.3. Lentiviral Infection and Generation of Stable Cell Lines

The shRNA-mediated lentivirus specific to human FARSA (FARSA^KD^), the overexpressed lentivirus for human FARSA (FARSA^OE^), and the corresponding control lentiviruses for FARSA^KD^ (FARSA^KD-Con^) and FARSA^OE^ (FARSA^OE-Con^) were purchased from Genechem (Shanghai, China). MCL cells were infected with lentiviruses according to the manufacturer’s instructions. The lentiviral-transduced cells were further selected with puromycin (2 μg/mL) for 7–10 days.

### 4.4. RNA Isolation and qRT-PCR

Total RNAs were isolated from cells using Trizol reagent and an RNA MiniPrep Plus kit (Zymo Research, Irvine, CA, USA) according to the manufacturer’s instructions, followed by qRT-PCR using a One Step SYBR PrimeScript PLUS RT-PCR kit (Takara, Kusatsu, Japan). The dissociation curve analysis was used to confirm the specificity of amplification for each gene and the absence of primer dimer. Each sample was performed in triplicate. The relative expression of each gene was normalized to the *ACTB* gene by the method of 2^−ΔΔCt^. The involved primers are indicated in Supplementary Table S1.

### 4.5. Immunoblotting and Semi-Quantitative Analysis

Cells were lysed with RIPA buffer (Beyotime, Shanghai, China). Equal amounts of proteins were separated on 8–12% SDS-PAGE gels according to different molecular weights, and then transferred onto polyvinylidene fluoride membranes (Millipore, Stafford, VA, USA). The membranes were then blocked with 5% bovine serum albumin, followed by incubation with primary and secondary antibodies. Proteins were visualized using an ECL kit (Bio-Rad, Hercules, CA, USA). Immunoblots were further subjected to semi-quantitative analysis using ImageJ software (National Institute of Health, Bethesda, MD, USA). The relative expression of target proteins was normalized to β-actin.

The following antibodies were used for immunoblots: FOXO1 (ab52857) and RAG1(ab172637) from Abcam (Boston, MA, USA); AKT (#9272), p-AKT (#9271), β-actin (#3700) as well as anti-mouse and anti-rabbit secondary antibodies from Cell Signaling (Danvers, MA, USA).

### 4.6. Cell Proliferation Assay

Cell proliferation was evaluated using a BeyoClick™ EdU Cell Proliferation Kit (Beyotime, Shanghai, China) according to the manufacturer’s instructions. Briefly, cells were seeded at a density of 1 × 10^6^ cells/mL in a 6-well plate. After 48 h, Edu (10 μM) was added to each well and incubated at 37 °C for 2 h. Cells were then collected followed by 4% paraformaldehyde fixation, permeabilization with 0.1% Triton X-100, and a 30 min exposure to click reaction cocktail. Staining cells were analyzed on a CytoFLEX flow cytometer (Beckman Coulter, Brea, CA, USA). The percentage of EdU-positive cells was defined as the proliferation rate.

### 4.7. Cell Cycle and Cell Apoptosis Assays

PI/RNase Staining Buffer and PE Annexin V Apoptosis Detection Kit (BD Biosciences, San Jose, CA, USA) were used to detect cell cycle and cell apoptosis, respectively. Staining cells were analyzed on a CytoFLEX flow cytometer (Beckman Coulter, Brea, CA, USA). All flow cytometry data were analyzed using FlowJo software (FlowJo^TM^, Ashland, OR, USA).

### 4.8. RNA-Seq Profiling and Analysis

Total RNAs were isolated from the FARSA^KD^ and FARSA^KD-Con^ Jeko cells (n = 2/group) to generate RNA libraries, which were sequenced on an Illumina HiSeq platform (Novegene, Tianjin, China). Genes with adjusted *p* value < 0.05 or the absolute log2-fold change >0 were considered as DEGs. The obtained DEGs were further used to draw the volcano map and heatmap. The RNA-seq raw data can be found in the Sequence Read Archive (SRA). The BioProject accession number is PRJNA780334, and the SRA records will be accessible at https://www.ncbi.nlm.nih.gov/sra/PRJNA780334 (released on 31 December 2022) upon publication.

### 4.9. GO Terms and Pathway Enrichment Analysis

GO- and KEGG-enrichment analyses of the obtained DEGs were carried out using the clusterProfiler R package. The adjusted *p* value < 0.05 was considered as statistically significant. The online AmiGO2 software (http://amigo.geneontology.org/amigo/landing, accessed on 26 November 2021) was further utilized to classify the significant GO terms into sub-categories, which were manually created if GO terms were classified into the same biological pathway or activity.

GSEA was performed to identify whether a set of genes in a specific pathway shows significant differences between two groups. Local version of GSEA analysis tool (http://www.broadinstitute.org/gsea/index.jsp, accessed on 12 November 2021) was used.

### 4.10. Statistical Analysis

Data are presented as mean ± standard error of mean (SEM) or standard deviation (SD). Differences between two groups were determined by the Student *t* test, where * *p* < 0.05 and ** *p* < 0.01 were considered statistically significant. All experiments and assays were repeated at least three times and performed in duplicate or triplicate.

## Data Availability

All data and materials in the current study are included in this paper and Supplementary Information.

## Supplementary Materials

The supporting information can be downloaded at: https://www.mdpi.com/article/10.3390/ijms24021608/s1.

## Abbreviations

aaRS(s)	aminoacyl-transfer RNA synthetase(s)
ATF5	activating transcription factor 5
BTZ	Bortezomib
CCNA2	cyclin A2
CCND1	cyclin D1
CDK4/6	cyclin dependent kinase 4/6
CDKN1A/2A	cyclin dependent kinase inhibitor 1A/2A
cyto	cytoplasmic
DEGs	differentially expressed genes
E2F1	E2F transcription factor 1
EIF4G1	eukaryotic translation initiation factor 4 gamma 1
EPRS1	glutamyl-prolyl-tRNA synthetase 1
FARS/PheRS	phenylalanyl-tRNA synthetase
FARSA	phenylalanyl-tRNA synthetase subunit alpha
FARSB	phenylalanyl-tRNA synthetase subunit beta
FHL1	four and a half LIM domains 1
FOXO	forkhead box O
GARS1	glycyl-tRNA synthetase 1
GO	Gene Ontology
GSEA	Gene Set Enrichment Analysis
IARS1	isoleucyl-tRNA synthetase 1
KEGG	Kyoto Encyclopedia of Genes and Genomes
KRAS	KRAS proto-oncogene
MAP2K1	MAPK kinase 1
MAPK1	mitogen-activated protein kinase 1
MCL	mantle cell lymphoma
mTORC1	mammalian target of rapamycin complex 1
MYC	MYC proto-oncogene, bHLH transcription factor
NHLs	non-Hodgkin’s lymphomas
p-AKT	phosphorylated AKT
PIK3CA/B	phosphatidylinositol-4,5-bisphosphaste 3-kinase catalytic subunit alpha/beta
qRT-PCR	quantitative real-time PCR
RAG1	recombination activating 1
RNA-seq	RNA sequencing
SD	standard deviation
SEM	standard error of mean
STN	Sotrastaurin
TARS1	threonyl-tRNA synthetase 1
TCGA	Cancer Genome Atlas
TERT	telomerase reverse transcriptase
tRNA	transfer RNA
WARS1	tryptophanyl-tRNA synthetase 1
