# Supplementary figures and images for: First Report of FARSA in the Regulation of Cell Cycle and Survival in Mantle Cell Lymphoma Cells via PI3K-AKT and FOXO1-RAG1 Axes
Source: Int J Mol Sci. 2023 Jan 13;24(2):1608. doi: 10.3390/ijms24021608 (PMC9865697; doi:10.3390/ijms24021608)

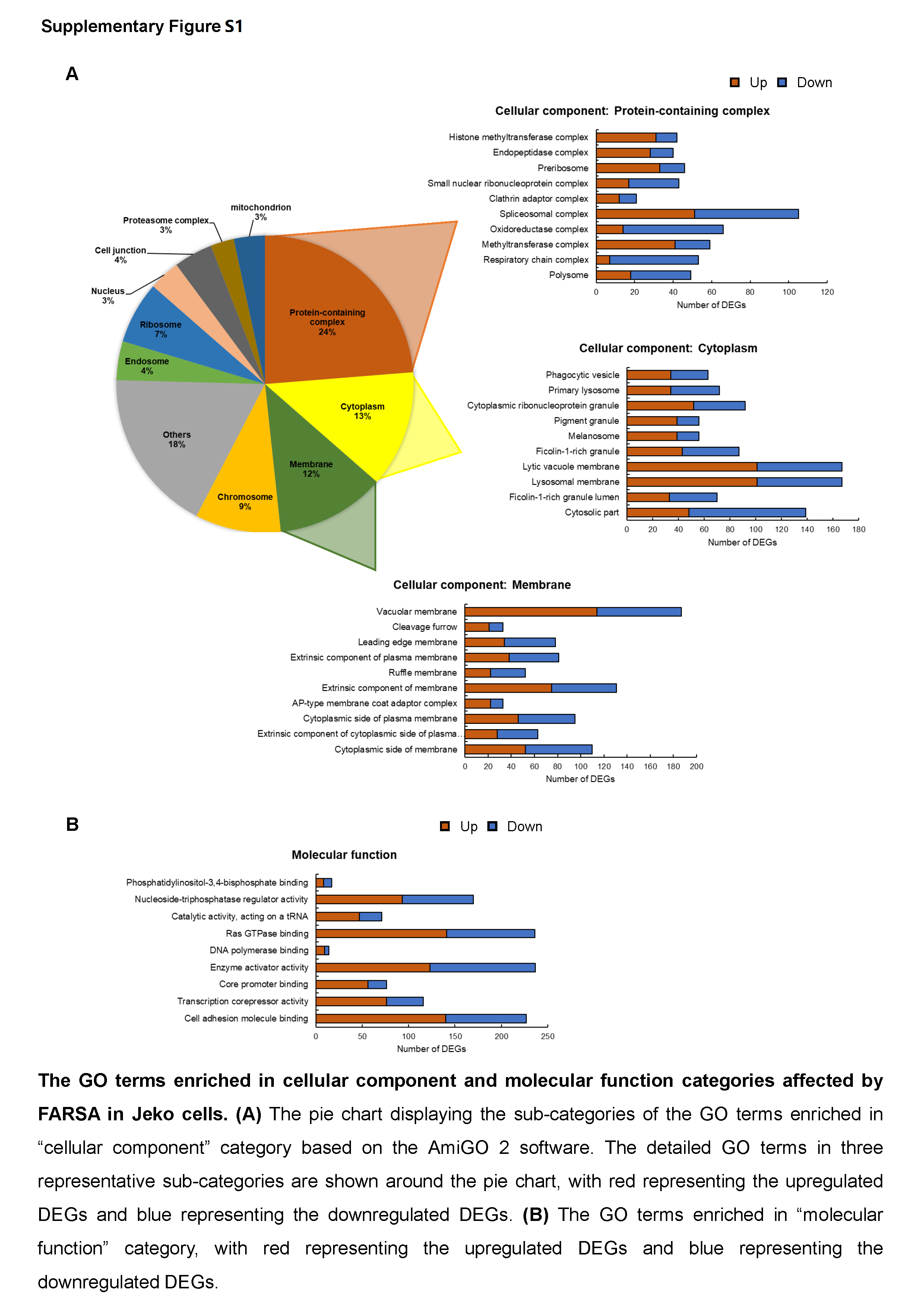

Supplement: Supplementary file 1 [file ijms-24-01608-s001.zip › Figure S1.tiff]

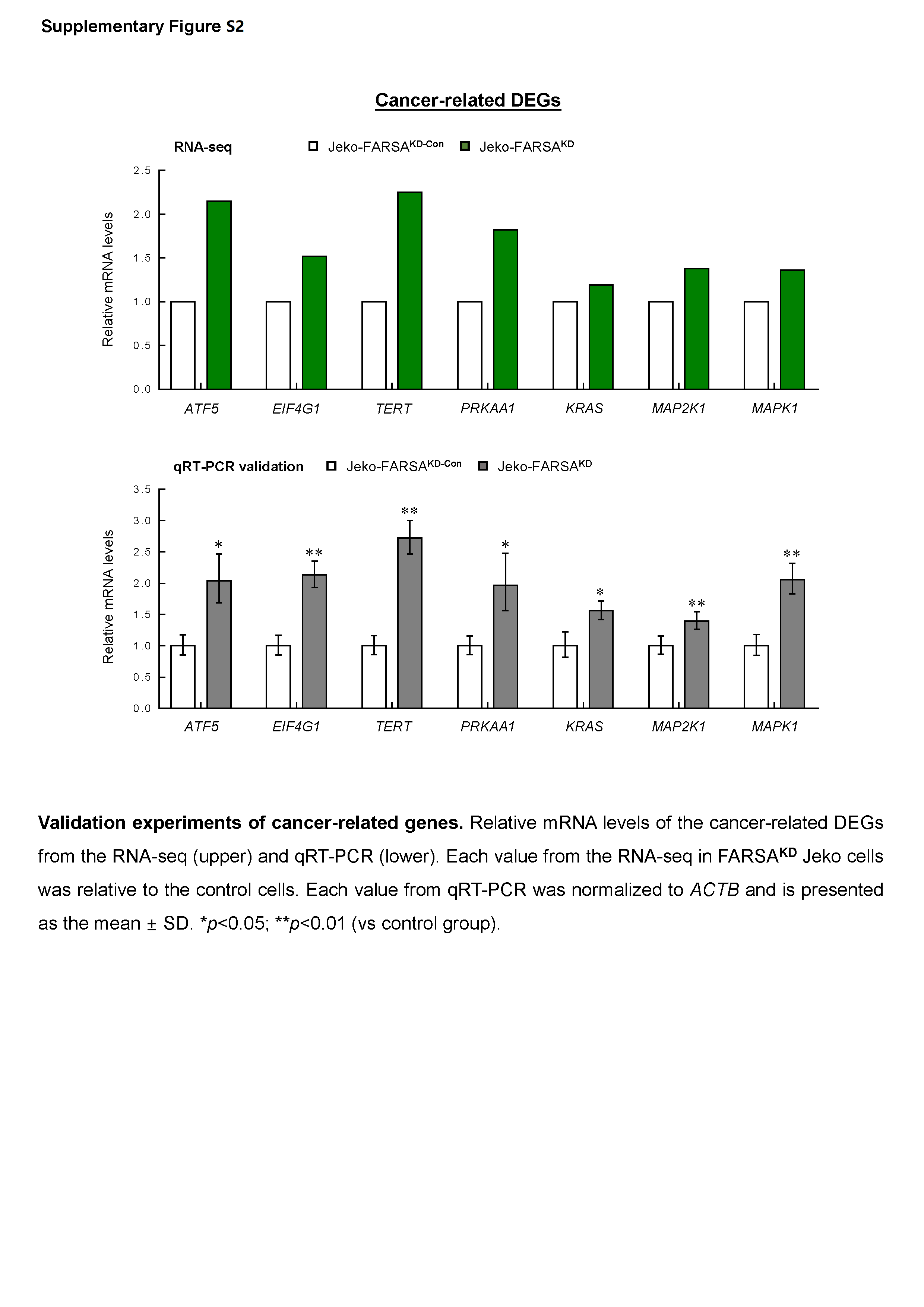

Supplement: Supplementary file 1 [file ijms-24-01608-s001.zip › Figure S2.tiff]
